# Future Mobile Device Usage, Requirements, and Expectations of Physicians in German University Hospitals: Web-Based Survey

**DOI:** 10.2196/23955

**Published:** 2020-12-21

**Authors:** Oliver Maassen, Sebastian Fritsch, Julia Gantner, Saskia Deffge, Julian Kunze, Gernot Marx, Johannes Bickenbach

**Affiliations:** 1 Department of Intensive Care Medicine University Hospital RWTH Aachen Aachen Germany; 2 SMITH Consortium of the German Medical Informatics Initiative Leipzig Germany; 3 Institute of Medical Statistics Informatics and Data Science Jena University Hospital Jena Germany

**Keywords:** mobile devices, mobile applications, apps, mHealth, smartphones, tablets, device usage, requirements, expectations, hospital, working equipment

## Abstract

**Background:**

The use of mobile devices in hospital care constantly increases. However, smartphones and tablets have not yet widely become official working equipment in medical care. Meanwhile, the parallel use of private and official devices in hospitals is common. Medical staff use smartphones and tablets in a growing number of ways. This mixture of devices and how they can be used is a challenge to persons in charge of defining strategies and rules for the usage of mobile devices in hospital care.

**Objective:**

Therefore, we aimed to examine the status quo of physicians’ mobile device usage and concrete requirements and their future expectations of how mobile devices can be used.

**Methods:**

We performed a web-based survey among physicians in 8 German university hospitals from June to October 2019. The online survey was forwarded by hospital management personnel to physicians from all departments involved in patient care at the local sites.

**Results:**

A total of 303 physicians from almost all medical fields and work experience levels completed the web-based survey. The majority regarded a tablet (211/303, 69.6%) and a smartphone (177/303, 58.4%) as the ideal devices for their operational area. In practice, physicians are still predominantly using desktop computers during their worktime (mean percentage of worktime spent on a desktop computer: 56.8%; smartphone: 12.8%; tablet: 3.6%). Today, physicians use mobile devices for basic tasks such as oral (171/303, 56.4%) and written (118/303, 38.9%) communication and to look up dosages, diagnoses, and guidelines (194/303, 64.0%). Respondents are also willing to use mobile devices for more advanced applications such as an early warning system (224/303, 73.9%) and mobile electronic health records (211/303, 69.6%). We found a significant association between the technical affinity and the preference of device in medical care (χs2=53.84, *P*<.001) showing that with increasing self-reported technical affinity, the preference for smartphones and tablets increases compared to desktop computers.

**Conclusions:**

Physicians in German university hospitals have a high technical affinity and positive attitude toward the widespread implementation of mobile devices in clinical care. They are willing to use official mobile devices in clinical practice for basic and advanced mobile health uses. Thus, the reason for the low usage is not a lack of willingness of the potential users. Challenges that hinder the wider adoption of mobile devices might be regulatory, financial and organizational issues, and missing interoperability standards of clinical information systems, but also a shortage of areas of application in which workflows are adapted for (small) mobile devices.

## Introduction

The usage of mobile devices, especially smartphones, has substantially increased, up to 95% in nearly all age groups in Germany [1]. With amazing advancement in mobile computer technology and connectivity, mobile devices have already revolutionized communication [2] as well as social media, mobility, fitness tracking, and further mobile health (mHealth) technologies, thus offering the potential to innovate health care inside and outside of clinical settings. The improvement of self-management as well as the effectiveness of the use of mHealth in professional medicine has been proven for several medical conditions, for example, the management of different chronic diseases such as arterial hypertension, diabetes or coronary heart disease, but also the management of acute diseases such as cardiac arrest and stroke [2-5]. Some authors even see smartphones as portable, multifunctional tools with the potential to become “the new stethoscope for physicians [6].” Increasingly, patients and the general population are being encouraged to take responsibility for their own health by actively monitoring their physiological parameters with smartphones, apps, and fitness trackers [7-10].

A fundamental component of the operationalization of mHealth is the usage of mobile devices, especially smartphones and tablets, by patients or health care professionals. The central areas of utilization of mobile devices in hospitals are (1) oral and written communication [11-15]; (2) documentation, organization, and information [16-19]; (3) decision support, notifications, and alarms [20-23], (4) education and professional training [24-28]; and (5) self-monitoring by physicians [29]. Taken together, physicians use mobile devices to assure their own decisions in a clinical environment and to increase efficiency in their workplaces [30].

The benefits of the usage of mobile devices in health care is counterbalanced by problems such as the colonization of surfaces with harmful pathogens or the distraction of medical staff [31,32]. Even more relevant are regulatory and organizational barriers for the implementation of mHealth apps on mobile devices in hospitals, missing standards for the development of health apps, information safety issues, and privacy concerns [12,33,34]. Furthermore, the parallel use of private devices for professional and private purposes is common [6], and thus further impacts data protection and patients’ privacy.

Both the combination use of mobile devices privately and on duty, and the physician’s attitude toward its deployment for so many different areas of utilization, make a structured systematic overview of actual needs difficult. There are hardly any data on the use of mobile devices in hospitals for clinical applications. Therefore, we aim to evaluate the current usage of mobile devices of physicians in German university medical centers and to explore their opinion and perceived needs regarding mobile devices.

## Methods

### Study Design, Data Collection, and Recruitment

For the preparation of the survey questions, an unstructured exploratory interview was conducted with 3 junior and 3 senior physicians focusing on their requirements and perceptions toward mobile devices. The results were used to construct the questionnaire for the actual study.

The study was designed as an open web-based survey in 8 German university hospitals and conducted among physicians of all medical disciplines (Limesurvey). To prove the functionality of the survey and the clarity of the questions, a test run was sent to a small group of 25 anesthesiologists and critical care physicians in June 2019. Minor remarks and improvement recommendations were made and integrated in the final version of the online survey which was sent via email with a link to the survey that was valid for 19 weeks. Responsible contact persons in the respective hospitals forwarded the link to physicians in their hospitals. During the period of data collection, no bug fixes and content changes were made, and no unexpected events such as system failures or server downtime were observed. The local data protection officer and the local ethics committee were consulted and had no concerns regarding the study. The title page of the survey contained information regarding the length, the foreseen time for completion, and the purpose the questionnaire. Completion of the survey was taken as consent for scientific usage of the collected data.

The survey was divided into sections, one of which contained questions about mobile devices usage. Biographical questions were included in another section (see Multimedia Appendix 1 for the English version of the survey).

Different types of survey questions were prepared: closed-ended questions, open-ended questions, rating questions, Likert scale questions (4-point scale), multiple choice questions, and demographic questions. Most questions allowed the participants to give multiple answers. Answering questions was not compulsory, as we expected that mandatory answers increases the risk that participants do not complete the web-based survey. Only fully completed questionnaires were included in the analysis. It was taken into account that some items were not answered by all participants (indicated as no response) resulting in a variation in the total number of answers. The survey instructions stated that cordless telephones (such as digital enhanced cordless telecommunications known as DECT) without additional functions and digital message receivers are not included in the survey to keep a narrower definition of a mobile device.

### Statistical Analysis

As this study aimed to provide a general overview over physicians' attitudes and expectations toward mobile devices, data were predominantly analyzed with descriptive counts and proportions, applying significance tests only in a few selected cases. For nominal variables with a particularly large number of values (eg, medical discipline), values were summarized into broad categories, if possible. Data are given as absolute numbers or their percentages; summaries are given as median and as interquartile range for ordinal data and mean and standard deviation for continuous variables. Some of the survey items allowed for multiple responses (eg, choose all that apply), thus invalidating the use of classical chi-square testing to check for associations between those items. For significance testing of multiple response item associations, the nonparametric bootstrap variant of the simultaneous pairwise marginal independence test proposed by Bilder and Loughin [35] was used, which was implemented in the MRCV package (version 0.3-3) [36] in R (version 4.0.1). To test for associations of score variables with single-response items, the Kruskal-Wallis rank sum test was used.

The 33 medical disciplines of the participants were classified into 6 categories (see Multimedia Appendix 2, Table S1) to identify dependencies between the discipline categories and the survey answers. Furthermore, the mobile devices question group, which investigates the current usage, the needs, and requirements of physicians, was simplified. For the analysis, the tasks conducted with mobile devices in 5 fields of application in stationary hospital care (defined as all fields of inpatient care) were categorized as follows: (1) oral and written communication; (2) documentation, organization, and information desk; (3) decision support, notifications, and alarms; (4) Education and professional training; and (5) self-monitoring by physicians (see Table 1).

**Table 1:** Fields of application of mobile devices in stationary hospital care.

Categories	Functions
1. Oral/written communication	Official phone calls Official text messages (eg, SMS, messenger) Web conferences (eg, tumor conferences)
2. Documentation, organization, and information desk	Time scheduling and workflow support Mobile EHR^a^ to look up patient information and for medical documentation Written instruction and recording procedures and examinations Written inter/intraprofessional communication (doctors, nurses, therapists, consult requests) To look up dosages, diagnoses and guidelines (online/offline)
3. Decision support, notifications, and alarms	Alarming while monitoring of vital signs (Early) warning system to prevent adverse effects (eg, pharmacological interaction) Decision support and definition of therapies
4. Education and professional training	Education system for job training, education and professional training
5. Self-monitoring by physicians	Monitoring of own vital signs/motion analysis (eg, pedometer, energy consumption)

^a^EHR: electronic health record.

We developed a scoring system to analyze the participants’ attitude toward mobile devices by assigning positive values to answers indicating a positive attitude toward mobile devices (fully disagree=0; fully agree=3) and negative values to EHR answers indicating a negative attitude (fully disagree=0; fully agree=–3). These values were summed for each participant and stratified by age groups, by medical disciplines, and by technical affinity.

## Results

### Demographic and Professional Characteristics

In total, 303 physicians with a mean clinical work experience of 12.7 years completed the survey. The full demographic and professional characteristics are given in Table 2. The participating physicians displayed a wide range of medical disciplines and the full range of discipline categories. The study population worked in all operational areas of the hospital, and physicians from all professional levels completed the web-based survey.

**Table 2:** Demographic and professional characteristics.

Characteristic		Value (n=303), n (%)	
**Age range (years)**			
	18-24		1 (0.3)
	25-34		98 (32.3)
	35-44		103 (34.0)
	45-54		69 (22.8)
	55-65		21 (6.9)
	>65		3 (1.0)
	No response		8 (2.6)
**Gender**			
	Female		121 (39.9)
	Male		173 (57.1)
	No response		9 (3.0)
**Current occupation**			
	Assistant physician		101 (33.3)
	Medical specialist		49 (16.2)
	Senior physician		108 (35.6)
	Clinic director		28 (9.2)
	Others		6 (2.0)
	No response		11 (3.6)
**Medical field/discipline**			
	Anesthesiology/intensive care medicine		75 (24.8)
	Internal medicine		53 (17.5)
	Pediatrics		25 (8.3)
	Surgery		22 (7.3)
	Neurology		14 (4.6)
	Dermatology		12 (4.0)
	Microbiology, virology, infectiology		10 (3.3)
	Psychiatry and psychotherapy		10 (3.3)
	Psychosomatic medicine and psychotherapy		8 (2.6)
	Neurosurgery		8 (2.6)
	Ophthalmology		7 (2.3)
	Pathology		7 (2.3)
	Otorhinolaryngology		5 (1.7)
	Child and adolescent psychiatry and psychotherapy		5 (1.7)
	Laboratory medicine		5 (1.7)
	Radiology		5 (1.7)
	Urology		5 (1.7)
	Other disciplines/specialization		43 (14.2)
**Predominant workplace**			
	Hospital ward		123 (40.6)
	Operating theatre		106 (35.0)
	Outpatient clinic		100 (33.0)
	Intensive care unit		89 (29.4)
	Office		50 (16.5)
	Laboratory		33 (10.9)
	Functional area		30 (9.9)
	Others		15 (5.0)
Clinical professional experience (years), mean (SD)		12.7 (9.3)	

### Mobile Device Usage: Devices and Operation Purposes

Almost all respondents had smartphones (294/303, 97.0%) and laptops or desktops (280/303, 92.4%) for private use outside the working environment, 61.7% (187/303) of the respondents used tablets, and 20.8% (63/303) used wearables such as smartwatches and fitness trackers privately.

In clinical daily routine, 71.0% of physicians (215/303) used mobile devices. The operational purposes of mobile devices in clinical practice are widespread (Table 3). Predominantly, mobile devices are used for basic functions such as looking up information, oral and written communication (text messages, emails), and time scheduling.

**Table 3:** For which operation purposes do you use mobile devices in your clinical routine?

Operation purposes	Value (n=303), n (%)
Phone calls	154 (50.8)
Text messages (eg, SMS, messenger)	130 (42.9)
Email communication	157 (51.8)
Look up of information	190 (62.7)
Mobile access to hospital information systems	57 (18.8)
Time scheduling	141 (46.5)
Private communication	107 (35.3)
Dictation of texts	18 (5.9)
Scientific work	79 (26.1)
Other	14 (4.6)

Almost 79% of all respondents (238/303, 78.6%) stated that they used private devices for official uses in daily clinical routine. The majority of the respondents stated that they used their private mobile device because they are not provided with an official mobile device in hospital care (146/303, 48.2%). Other reasons were the ability to work at home (137/303, 45.2%) and the fact that mobile work is only possible with their private mobile devices (105/303, 34.7%). Some respondents also stated that their private devices are better than the official device (85/303, 28.1%) and that private communication is only possible with their private mobile device (65/303, 21.5%).

### Perceived Ideal Device Versus Actual Time of Usage

**Figure 1:**
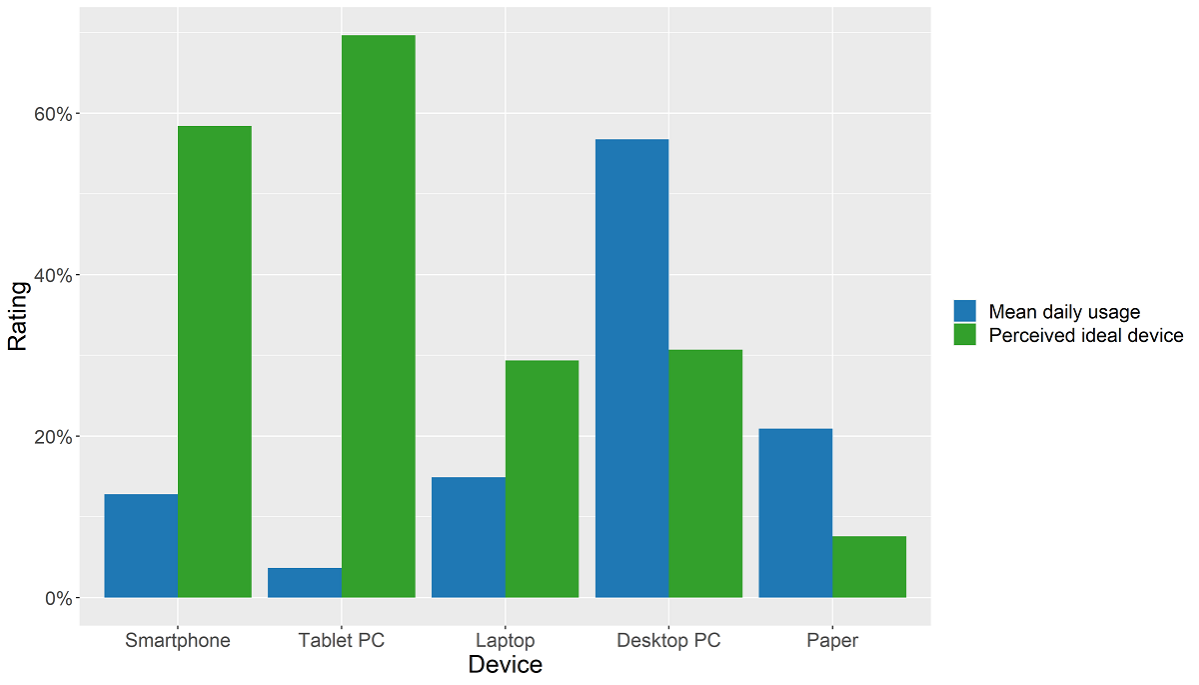
Percentage of respondents rating device as ideal device versus mean percentage of daily worktime used.

The vast majority of participating physicians rated tablets (211/303, 69.6%) and smartphones (177/303, 58.4%) as the most appropriate device for their area of application (Figure 1). A smaller group of respondents (93/303, 30.7%) ranked desktop computers as most appropriate, and a group of respondents (89/303, 29.4%) regarded laptops as the most suitable device for their work area. Still 23 respondents (23/303, 7.6%) rated paper as most suitable for their professional work. Respondents could choose more than one answer for this question, and the simultaneous pairwise marginal independence test revealed no significant differences in answers between the different age groups (χ_s_^2^=34.19, *P*=.052).

Contrasting their conception of ideal device usage, the physicians were asked to rate their actual time of usage of the devices during their daily total worktime. While most physicians rated smartphones and tablets as the perceived ideal device, in today’s clinical practice, desktop computers and paper are predominantly used (Figure 1).

### Personal Opinion About Mobile Devices

In another survey section, we asked about the personal opinion of physicians toward mobile devices in stationary hospital care. We inquired whether mobile devices are supportive tools, whether they should be implemented in stationary hospital care and whether they increase patient safety. Furthermore, we asked for data security and information safety concerns regarding mobile devices and whether respondents fear an increasing workload or increasing operational supervision. For analysis, the statements “rather applies” and “fully applies” were taken as agreement. Most respondents (276/303, 91.1%) agreed with the statement “A mobile device would support me in my work.” Moreover, most physicians wish for the area-wide implementation of mobile devices in stationary patient care (259/303, 85.5%).

**Figure 2:**
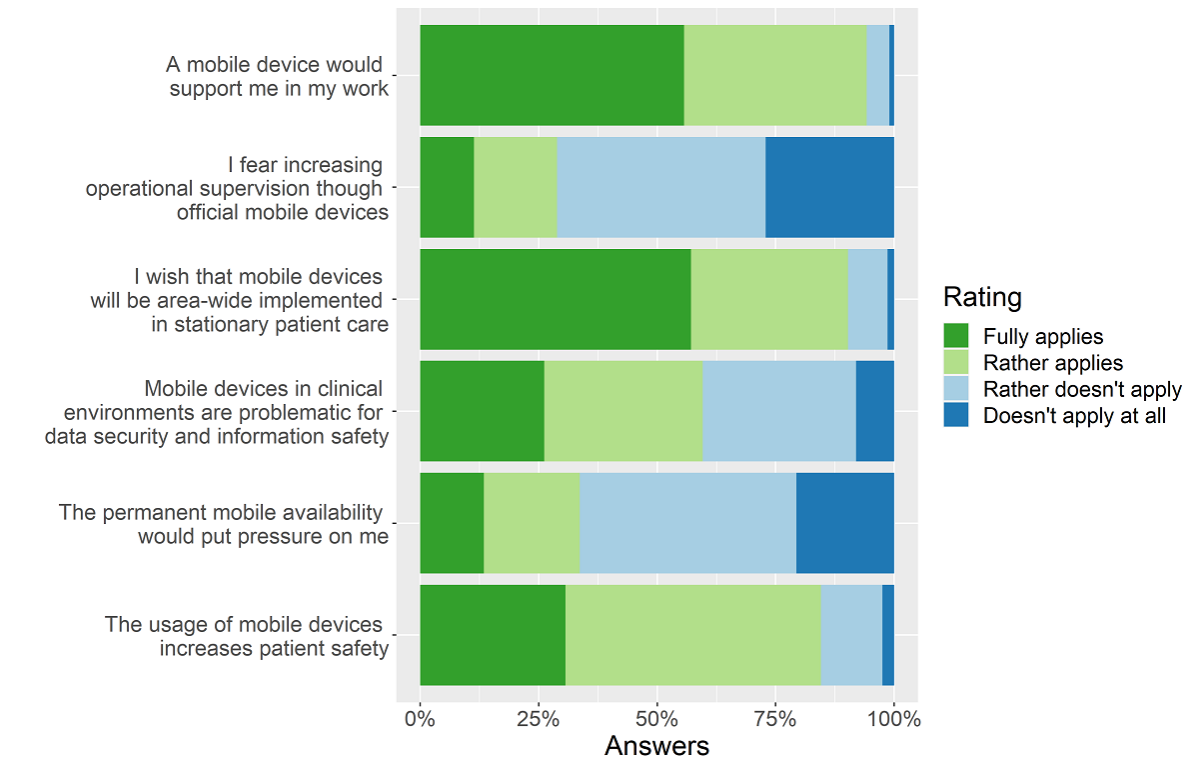
Personal opinion about mobile devices.

Regarding data security and information safety, a slight majority of 56.4% (171/303) of respondents expressed concerns. However, the large majority of 79.2% (240/303) of respondents agreed that the usage of mobile devices could increase patient safety (Figure 2).

In sum, most physicians rated mobile devices as useful and supportive tools that should be implemented in stationary hospital care.

**Figure 3:**
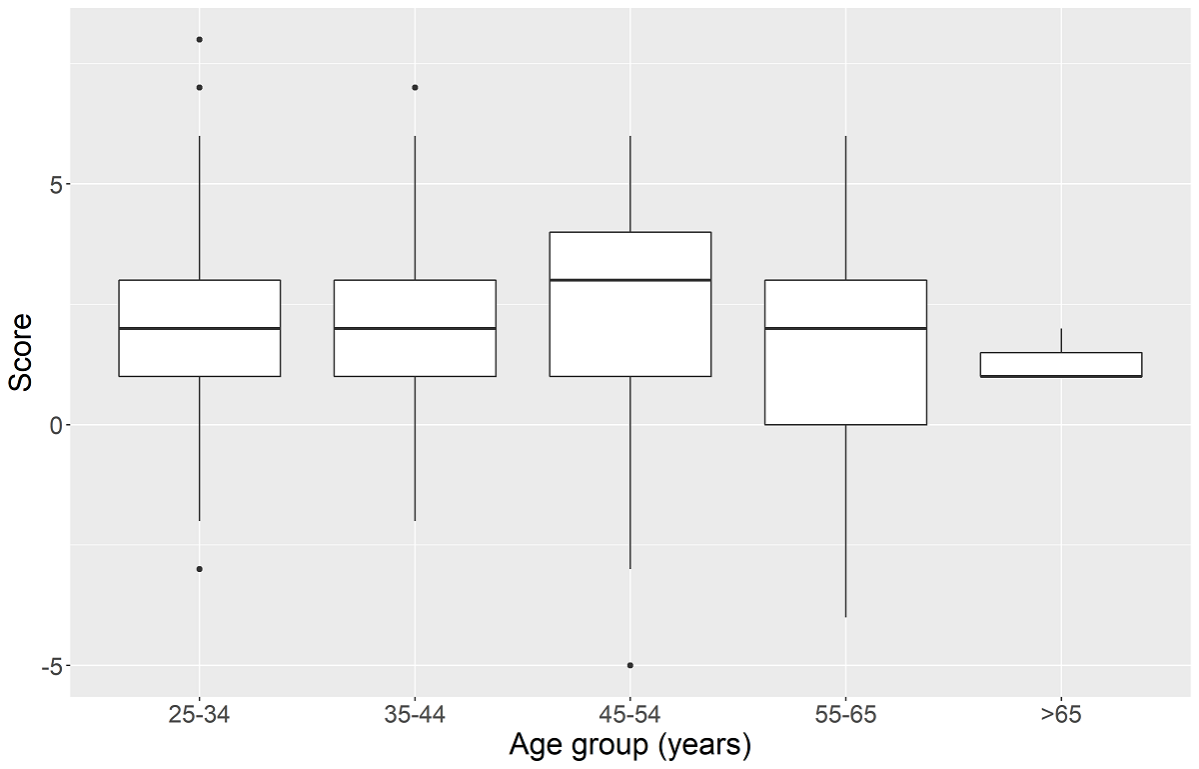
Attitude toward mobile devices stratified by age groups.

In general, a Kruskal-Wallis test did not show significant differences in the attitude toward mobile devices between age groups (*H*_5_=7.29, *P*=.20; Figure 3).

### Self-Reported Technical Affinity

Overall, the study population reported a high technical affinity level (mean 4 out of 5).

**Figure 4:**
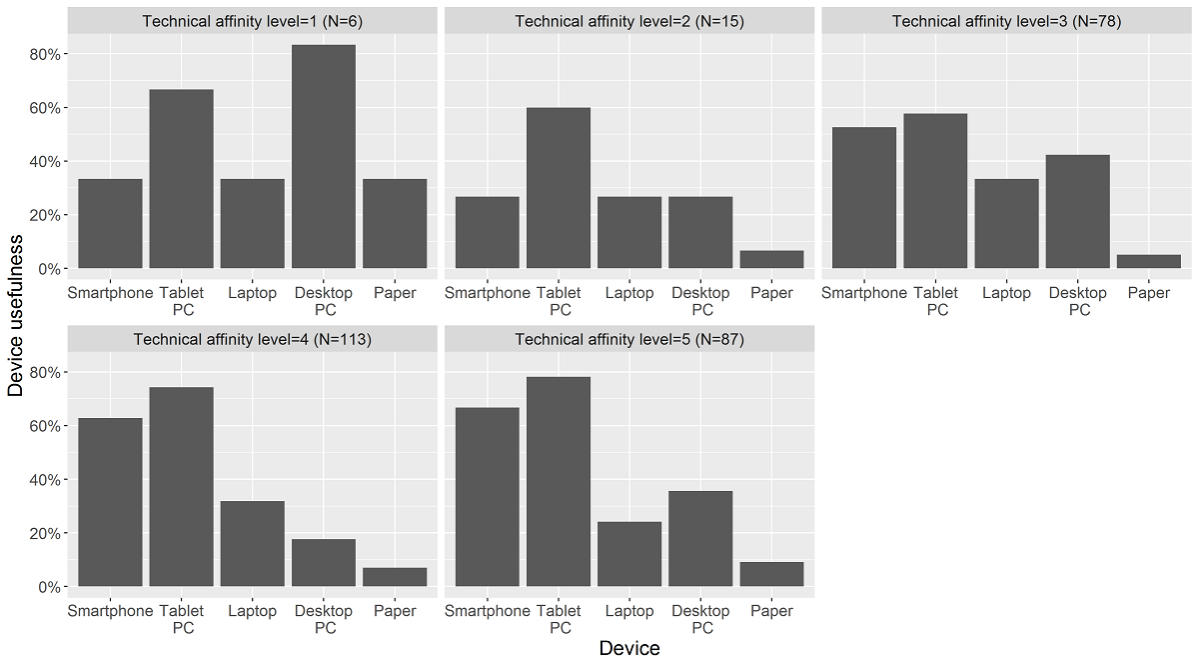
Device preference stratified by (self-reported) technical affinity level.

There was a significant association between technical affinity and the preference of device in medical care (χ_s_^2^=53.84, *P*<.001) showing that with increasing self-reported technical affinity the preference for smartphones and tablets increases compared to desktop computers (Figure 4).

**Figure 5:**
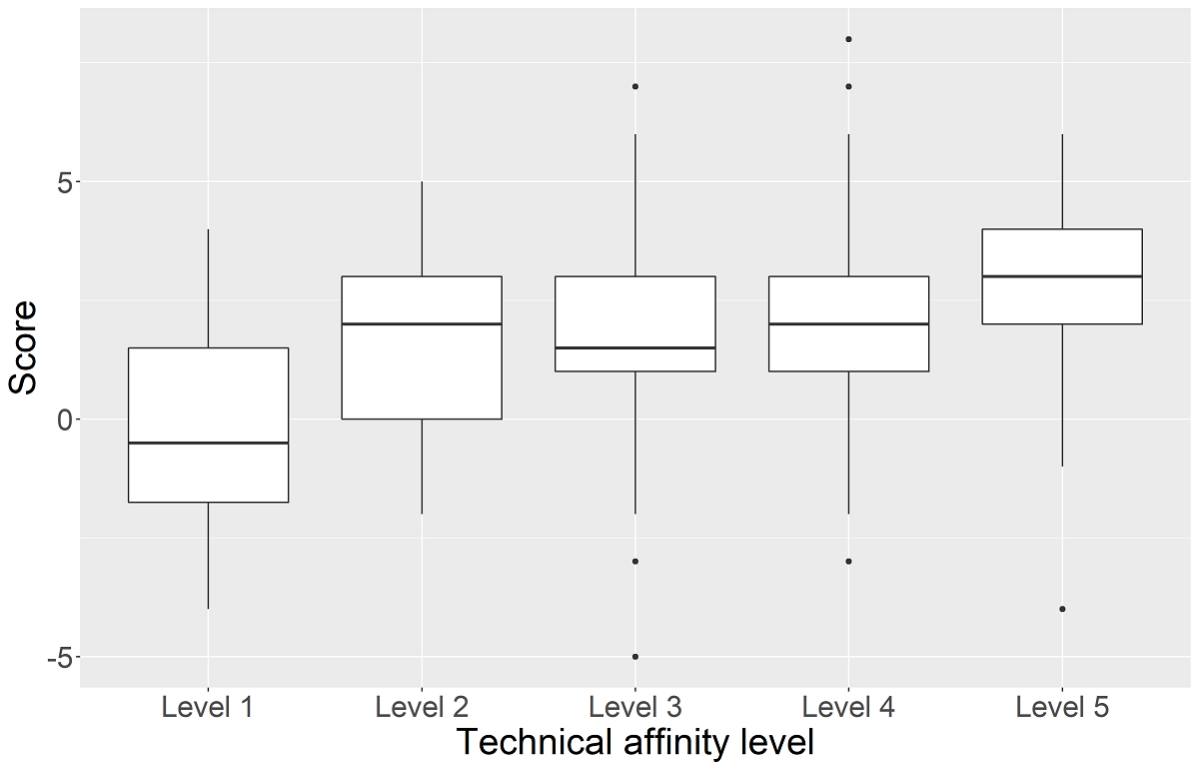
Attitude toward mobile devices stratified by (self-reported) technical affinity level.

The most optimistic respondents toward mobile devices were those who also had the highest self-reported technical affinity (Figure 5). With increasing technical affinity, the score for the attitude toward mobile devices increased (*H*_4_=17.31, *P*=.002).

### Fields of Application and Desired Uses of Mobile Devices in Medicine

**Figure 6:**
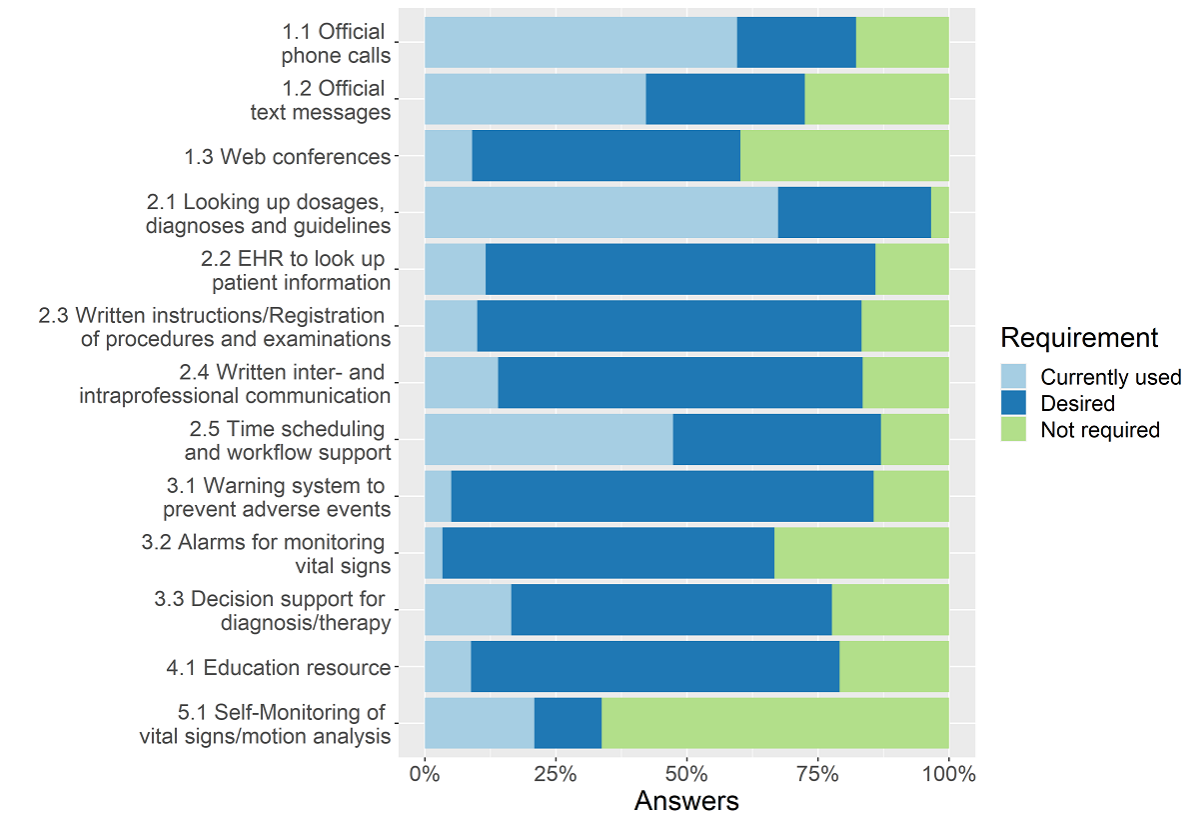
Fields of application and desired functions. EHR: electronic health record.

Finally, we examined the actual use of mobile devices and which functions the participants desired to use in stationary hospital care, if available. Five major fields of application of mobile devices could be identified (Table 1). Within these fields, the following 4 functions were the most commonly used mobile device apps by physicians: official phone calls (171/303, 56.4%); official text messages (118/303, 38.9%); looking up dosages, diagnoses, and guidelines (194/303, 64.0%); and time scheduling and workflow support (135/303, 44.6%). The most desired mobile device uses were for (early) warning systems to prevent adverse effects in hospital care (eg, pharmacological interactions; 224/303, 73.9%), for mobile EHR to look up patient information and for medical documentation (211/303, 69.6%), and for written instructions and recording procedures and examinations (206/303, 68.0%). The results can be found in Figure 6.

## Discussion

### Current Situation and Physicians’ Requirements

In today’s clinical practice, physicians are confronted with increasing amounts of patient information, information overload and information inaccessibility, endangering patient safety and potentially leading to fatal errors [37]. A substantial proportion of physicians use their private mobile devices for professional purposes, especially because they are not provided with official mobile devices for their work. Our survey results show that physicians expect that an official mobile device would support them in their work and increase patient safety. Most participants used mobile devices in hospitals for communication and organization (phone calls, text messages, time scheduling, and information), and thus, to increase the efficiency in their workplaces [30]. Among our study population, most physicians would prefer to use official devices but accept using their private mobile devices for professional purposes in stationary hospital care if there is no alternative.

### Potential Benefits for Physicians and Patient Care

Currently, physicians in hospital care spend a large amount of their worktime performing documentation in clinical information systems [38,39]. Therefore, physicians want to use mobile devices for documentation and recording procedures in these systems. Today, the tasks of documentation and recording procedures are most commonly performed with a desktop computer and cannot be performed in equal quality on a smaller mobile device. Thus, not every task in clinical care can be transferred from a desktop computer to a smartphone or a tablet without changing the process. Drews et al [16] examined the impact of the form factor of various mobile devices and desktop computers on the usability of EHRs; the authors concluded that even the largest form of a mobile device does not perform as well as a desktop computer for the usage of EHR. Consequently, the processes of documentation and recording procedures need to be changed before they can be performed in equal quality on a tablet or a smartphone in hospital care.

Smartphones and tablets at the point of care could support in-hospital physicians (eg, with functionalities such as taking notes via voice recognition for clinical documentation instead of keyboard-and-mouse interface) [40,41]. Payne et al [42] described the implementation of a smartphone-based system as a supporting system with automated speech recognition integrated in a commercial EHR; the mobile supporting system that was described has the potential to reduce the documentation burden of doctors and nurses, which is perceived as one of the big problems in today's health care [38,39]. We assume that physicians would benefit from mobile devices with automated speech recognition for documentation in and access to clinical information systems. Nevertheless, 48.2% (146/303) of all respondents are not provided with official mobile devices for these uses in hospital care and, as shown in Figure 6, less than 11% are already using a mobile EHR in our study population. As a precondition of continual documentation of patient information, mobile apps need to be integrated in central information systems in hospitals by using technical and semantic interoperability standards.

German hospitals are no exception in the rare use of mobile devices even for basic tasks, such as documentation in clinical information systems. However, physicians are open to an increasing use of more advanced mobile uses such as decision support systems on mobile devices in their clinical practice. Large proportions of physicians stated the wish to be able to use (early) warning systems for prevention of adverse effects, possibly harmful pharmacological interactions, smart monitoring of vital signs and decision support, and definition of therapies in clinical practice.

### Regulatory Challenges

The development of medical software such as mobile apps, especially those subject to new European Medical Device Regulation 2017/745, is a complex process including many obligations and requirements for manufacturers [43]. In addition to the regulatory legal requirements of the European Medical Device Regulation, health care professionals ask for the certification of health care apps [44].

The majority of physicians are aware that mobile devices may have implications on data security and information safety, while at the same time increasing patient safety. To ensure data security and information safety in hospitals, we suggest that apps that support health care professionals in performing complex and critical tasks should be installed and operated on official smartphones and tablets. For fulfilling information security standards, the devices should be provided and administrated through the hospital information technology departments. This could also help to reduce the potential legal grey area of using private mobile devices in the clinical environments for official purposes. Therefore, hospitals should implement a mobile device management process to safeguard the secure operation of mobile devices. Due to the increasing complexity of mobile device uses and increasing competencies of physicians in using mHealth apps, there is a need for a process to implement, teach, supervise, and evaluate clinical mHealth as well as mobile device and app competencies [24,45]. Finally, the added value of apps on mobile devices for physicians should be scientifically proven before being implemented in hospital care.

### Strengths and Limitations

This web-based survey covered the use of mobile devices for physicians in stationary hospital care in 8 university hospitals in different regions in Germany. As far as we know, it is the first survey covering this study population and evaluating the usage of, requirements for, and expectations toward mobile devices. University hospitals accommodate all medical disciplines and physicians involved in patient care, research, teaching, and training. Thus, a wide range of medical disciplines was covered by the respondents.

Usage of and requirements for mobile devices such as mobile (telemonitoring) apps for patients (self-monitoring and mobile self-reporting [46]) or diagnostic instruments connected to a mobile device (eg, iECG or handheld ultrasound [3]) are not the subject matter of this study. We also did not distinguish between official mobile devices as personalized or shared mobile devices.

A limitation of the recruitment method of the web-based survey is potential volunteer bias. The mean self-reported technical affinity of the study population was 4 out of 5, suggesting that most participating physicians had a relatively high affinity for technology, in general and in medical practice. Consequently, further research, using paper-based and personal-oral survey methods, is needed to reach physicians with a lower technical affinity.

### Conclusions

So far, the widespread use of official mobile devices such as smartphones and tablets has not become reality in stationary hospital care in German (university) hospitals. As long as physicians are predominantly using their private devices in clinical care, the usage of advanced apps with a deeper integration into the clinical information system infrastructure to support physicians remains uncertain.

Nevertheless, among the participating physicians of German university hospitals, technical affinity is high, and they have a very positive attitude toward mobile devices for clinical care. With our results, we demonstrated that the majority of the participating physicians used mobile devices for basic functionalities in hospital care. Although most physicians would prefer to work with mobile devices for documentation, writing instructions, and recording procedures in clinical information systems, a desktop computer is generally used for these tasks. Furthermore, physicians are willing to use their mobile devices for more progressive uses such as decision support systems or early warning systems. Thus, reasons for the low usage of official mobile devices in German hospitals are not the potential users, but rather regulatory, financial and organizational challenges, and missing interoperability standards.

While most physicians think that mobile devices would support their work and increase patient safety, they also mentioned concerns regarding data security and information safety.

## Appendix

Multimedia Appendix 1 Survey on the usage of mobile devices in stationary hospital care.

Multimedia Appendix 2 Categories of medical disciplines.

## Abbreviations

EHR: electronic health record
mHealth: mobile health
