# Supplementary material for: Future Mobile Device Usage, Requirements, and Expectations of Physicians in German University Hospitals: Web-Based Survey
Source: J Med Internet Res. 2020 Dec 21;22(12):e23955. doi: 10.2196/23955 (PMC7781804; doi:10.2196/23955)
Supplement: Multimedia Appendix 1 [file jmir_v22i12e23955_app1.doc]

# Multimedia Appendix

## “Future Mobile Device Usage, Requirements, and Expectations of Physicians in German University hospitals: Web-Based Survey”

**Survey on the usage of mobile devices in stationary hospital care**

The digitalisation of the health care system is a major challenge for all stakeholders involved. This concerns everyday aspects, such as the increasing use of mobile devices in clinical routine as well as developments with the potential of fundamentally transforming clinical practice, e.g. the use of artificial intelligence in medicine, like "big data analysis".

The attitude of physicians working in inpatient care towards these topics is still largely unexplored. Hence, we would like to request you as physicians to participate in the following survey. We would be grateful for your support. The survey includes 24 questions and requires 10 minutes to complete.

**Question Group 1 – Questions about mobile devices**

Usage of mobile devices in healthcare

The digitalisation of the health care system is progressing inexorably. In inpatient care, more and more data on diagnostics and therapy are being collected and documented in databases. In addition, numerous processes in German hospitals are supported by clinical information systems and documented in them. At the same time, the need to be able to access relevant information digitally and on mobile devices is also increasing.

This opens up a wide range of application for mobile devices such as smartphones and tablet PCs in inpatient care, which however are not yet being used on a nationwide basis. Plus, there is insufficient information on the willingness of hospital doctors to use mobile devices during their work and the requirements they demand on such devices.

Note: Cordless telephones without additional functions and digital message receivers are not included in this survey.

**1) Do you use mobile devices in your clinical daily routine?**

Please select only one of the following answers:

 Yes

 No

 No response

**2) If yes, what purpose do you use mobile devices for in your clinical routine?**

Please select all applicable answers:

 Phone calls

 Text messages (e.g. SMS, Messenger)

 Email communication

 Looking up of information

 Mobile access to hospital information systems

 Time scheduling

 Private communication

 Dictation of texts

 Scientific work

 Other________________________________________

**3) Do you also use private devices in your clinical daily routine?**

Please select only one of the following answers:

 Yes

 No

 No response

**4) If yes, why do you use private devices for official use?**

Please select all applicable answers

 Working from home

 Private device better than the official device

 No official devices provided

 Mobile work only possible with a private device

 Private communication at work only possible with private device

 Other________________________________________

**5) Would you like to use an official mobile device for private purposes?**

Please select only one of the following answers:

 Yes

 No

 I already use an official device for private purposes

 No response

**6) Would you like to use a private mobile device for official use?**

Please select only one of the following answers:

 Yes

 No

 I already use my private device for official purposes

 No response

**Question about the official usage in clinical stationary hospital care:**

**7) Please rate the extent to which you use the devices mentioned below during working hours (in percentage):**

______Smartphone

______Tablet PC

______Laptop

______Desktop PC

______Paper (medical documentation, reports, written diagnosis)

______Other

**Fields of application of mobile devices in clinical stationary care:**

**8) What are your personal fields of application of mobile devices in stationary hospital care?**

|  |  | I use | I wish | No necessity | No response |
| --- | --- | --- | --- | --- | --- |
| **8.1** | **Official phone calls** |  |  |  |  |
| **8.2** | **Official text messages (SMS, text messenger etc.)** |  |  |  |  |
| **8.3** | **Time scheduling and workflow support** |  |  |  |  |
| **8.4** | **Looking up dosages, diagnosis and guidelines** |  |  |  |  |
| **8.5** | **Mobile EHR to look up patient information and medical documentation** |  |  |  |  |
| **8.6** | **Written instruction and registration of procedures and examinations** |  |  |  |  |
| **8.7** | **Written inter-/ intraprofessional communication (doctors, Nurses, therapists, consult requests)** |  |  |  |  |
| **8.8** | **Alarming while monitoring of vital signs** |  |  |  |  |
| **8.9** | **(Early) warning system to prevent adverse effects (e.g. pharmacological interaction)** |  |  |  |  |
| **8.10** | **Decision support in diagnostics and definition of therapies** |  |  |  |  |
| **8.11** | **Education system for job training, education and professional training** |  |  |  |  |
| **8.12** | **Monitoring of own vital signs/ motion analysis (e.g. pedometer, energy consumption)** |  |  |  |  |
| **8.13** | **Web conferences (e.g. tumor conferences)** |  |  |  |  |

**9) Which device do you rate most appropriate for your application area?**

 Smartphone

 Tablet PC

 Laptop

 Desktop PC

 Paper

 Other________________________________________

**Personal opinion about mobile devices in clinical stationary care:**

**10) Please rate to what extend you agree with the statements on mobile devices in hospital care below.**

|  |  | Doesn’t apply at all | Rather doesn’t apply | Rather applies | Fully applies | No response |
| --- | --- | --- | --- | --- | --- | --- |
| **10.1** | **A mobile device would support me in my work** |  |  |  |  |  |
| **10.2** | **Mobile devices in clinical environments are problematic for data security and information safety** |  |  |  |  |  |
| **10.3** | **I wish that mobile devices will be area-wide implemented in stationary patient care** |  |  |  |  |  |
| **10.4** | **The permanent mobile availability would put pressure on me** |  |  |  |  |  |
| **10.5** | **I fear increasing operational supervision though official mobile devices** |  |  |  |  |  |
| **10.6** | **The usage of mobile devices increases patient safety** |  |  |  |  |  |

**Question group 3 – Biographical questions**

**Finally, we would like to ask you to answer the following biographical questions.**

**11. Your Age**

Please select one of the following answers:

 18-24 years

 25-34 years

 35-44 years

 45-54 years

 54-65 years

 >65 years

 No response

**12. Your Gender**

Please select one of the following answers:

 Female

 Male

 Diverse

 No response

**13. Your current occupation**

Please select one of the following answers:

 Assistant physician

 Medical specialist

 Senior Physician

 Clinic director

 Other________________________________________

 No response

**14. Your medical field/discipline**

Please select all applicable answers:

 Anaesthesiology/intensive care medicine

 Anatomy

 Biochemistry

 Child and adolescent psychiatry and psychotherapy

 Dermatology

 Forensic medicine

 General medicine

 Gynaecology

 Human genetics

 Hygiene and environmental medicine

 Internal medicine

 Laboratory medicine

 Microbiology, virology, infectiology

 Neurology

 Neurosurgery

 Nuclear medicine

 Occupational medicine

 Ophthalmology

 Oral and maxillofacial surgery

 Otolaryngology

 Paediatrics

 Pathology

 Pharmacology

 Physical and rehabilitative medicine

 Psychology

 Psychosomatic medicine

 Public healthcare

 Radiology

 Radiotherapy

 Surgery

 Transfusion medicine

 Urology

 Venereology

 Other disciplines/ specialization________________________________________

**15. Your predominant workplace**

Please select all applicable answers:

 Operating Theatre

 Hospital Ward

 Outpatient Clinic

 ICU Ward

 Functional Area

 Laboratory

 Office

 Other________________________________________

**16. How many years have you been clinically active for?**

Please enter your answer here:

**________years**

**Questions about the private use of laptops, PCs, mobile devices (tablets, smartphones) and wearables**

**17. Which devices do you use privately?**

Please select all applicable answers:

 Smartphone

 Tablet-PC (e.g. Apple iPad, Samsung Galaxy Tab, etc.)

 Laptop/ PC

 Wearables (e.g. Apple Watch, Garmin Vivo, Fitbit, etc.)

 Other ________________________________________

**18. How pronounced would you rate your affinity to engineering and information technology?**

**Scale 1 - 5 (1 = not pronounced; 5 = very pronounced)**

Please select one of the following answers:

 1

 2

 3

 4

 5

**19. Here is space for your remarks and comments on this survey:**

Please enter your answer here:

**__________________________________________________________________________________**

**We would like to thank you very much for participating in this survey. You can now close the window of your Internet browser.**
