# Supplementary material for: Future Mobile Device Usage, Requirements, and Expectations of Physicians in German University Hospitals: Web-Based Survey
Source: J Med Internet Res. 2020 Dec 21;22(12):e23955. doi: 10.2196/23955 (PMC7781804; doi:10.2196/23955)
Supplement: Multimedia Appendix 2 [file jmir_v22i12e23955_app2.doc]

# Multimedia Appendix

## “Future Mobile Device Usage, Requirements, and Expectations of Physicians in German University hospitals: Web-Based Survey”

**Table S1**: Categories of medical disciplines

| **Category name** | **Medical disciplines** |
| --- | --- |
| 1. Anaesthesia and intensive care medicine | - Anaesthesia/ intensive care medicine  - Emergency care  - Palliative care |
| 2. Surgical disciplines | - Ophthalmology  - Surgery  - Gynecology  - Otorhinolaryngology  - Oral and maxillofacial surgery  - Neurosurgery  - Urology  - Orthopedics and trauma surgery  - Dentistry |
| 3. Internal and conservative disciplines | - Internal medicine  - General medicine  - Occupational medicine  - Dermatology  - Pediatrics  - Physical and rehabilitative medicine  - Forensic medicine |
| 4. Neurological and psychiatric disciplines | - Child and adolescent psychiatry and psychotherapy  - Neurology  - Psychology  - Psychosomatic medicine |
| 5. Theoretical disciplines | - Pharmacology  - Biochemistry |
| 6. Diagnostic disciplines | - Radiology  - Laboratory medicine  - Microbiology, virology, infectiology  - Pathology  - Nuclear medicine  - Radiotherapy  - Human genetics  - Hygiene and environmental medicine |
